# Observer Design for a Core Circadian Rhythm Network

**DOI:** 10.1155/2014/476912

**Published:** 2014-07-08

**Authors:** Yuhuan Zhang

**Affiliations:** School of Mathematics and Information Sciences, Henan University, Kaifeng 475004, China

## Abstract

The paper investigates the observer design for a core circadian rhythm network in *Drosophila* and *Neurospora*. Based on the constructed highly nonlinear differential equation model and the recently proposed graphical approach, we design a rather simple observer for the circadian rhythm oscillator, which can well track the state of the original system for various input signals. Numerical simulations show the effectiveness of the designed observer. Potential applications of the related investigations include the real-world control and experimental design of the related biological networks.

## 1. Introduction

Controllability, observability, and stability are typical problems of dynamical systems [1, 2]. Suppose we have a dynamical system with the following form:

(1) $$\begin{array}{c} \frac{dx}{dt}=f\left(t,x\left(t\right),u\left(t\right)\right), \end{array}$$

where *x*(*t*) ∈ *R* ^*N*^ is the state vector of the system and *u*(*t*) ∈ *R* ^*M*^ is the input vector. System (1) is said to be observable, if we can find a function

(2) $$\begin{array}{c} y\left(t\right)=h\left(t,x\left(t\right),u\left(t\right)\right), \end{array}$$

from which one can determine all the state variables of system (1). Here, *y*(*t*) ∈ *R* ^*P*^ depends on *t*, a set of the system's state *x*(*t*) and the external input *u*(*t*) [1–6].

For *f*(*t*, *x*(*t*), *u*(*t*)) with polynomial or rational expressions, existing results have reported that system (1) is observable if the Jacobian matrix *J* = [*J*_*ij*_]_*NP*×*N*_ has full rank [3, 4], where *J*_*ij*_ is the Lie derivative of the output function *h*(*t*, *x*(*t*), *u*(*t*)).

Recently, Liu et al. [4] proposed a graphical approach to reduce the observability problem to a property of the inference diagram of a system. The inference diagram is based on the dynamical equation of the system. If *x*_*j*_ appears in *x*_*i*_'s differential equation, then there is a link from *x*_*i*_ to *x*_*j*_ in the inference diagram. The inference diagram can be decomposed into some strongly connected component (SCC). Those SCCs without incoming edges are called root SCCs. Liu et al. [4] reported that the number of the root SCCs provides the lower bound of the monitored state variables in *y*(*t*). Furthermore, for many nonlinear systems, they declared that the number of the root SCCs provides not only necessary but also sufficient numbers of state variables to realize observability. That is, if the inference diagram of a system has *k* root SCCs, then one may only need to select *k* state variables from different root SCCs, and the system will be observable through monitoring these *k* state variables.

Biological systems are typical nonlinear systems [7–23]. Observer design for biological systems has important real-world implications. For example, through monitoring a few state variables of a complex biological system, if we can infer the state of the whole system, then lots of resources can be saved [4]. Circadian rhythms are typical biological phenomenon, which display endogenous, entrainable oscillations with a period that lasts approximately 24 hours. Circadian rhythms are widely in existence in various plants and animals [12], which are controlled by biomolecular networks. Circadian rhythms have been extensively investigated during the last decades [18–23]. For example, in 1995, Goldbeter established a mathematical model for the circadian rhythms in the* Drosophila* [11]. In 1999, Leloup et al. established a model for the circadian rhythms in the* Drosophila* and* Neurospora* [13]. In 2002, Gonze and coauthors [7] investigated the deterministic and stochastic dynamics in a core circadian rhythm network. They found that the core network can display roughly the same circadian oscillations under both deterministic and stochastic descriptions.

In this paper, based on the graphical approach introduced by Liu et al. [4], for the core circadian rhythm network [7] in* Drosophila* and* Neurospora*, we design some simple observers for the network. Based on the Lyapunov stability theory, we theoretically verify the correctness of the designed observers. Finally, numerical simulations show the effectiveness of the designed observers for various input signals. The rest of the paper is organized as follows. In Section 2, we briefly introduce the mathematical model for the core circadian rhythm network. Observers will be designed in Section 3. We perform numerical simulations in Section 4. Discussions and some concluding remarks will be in Section 5.

## 2. The Core Circadian Rhythm Model

**Figure 1:**
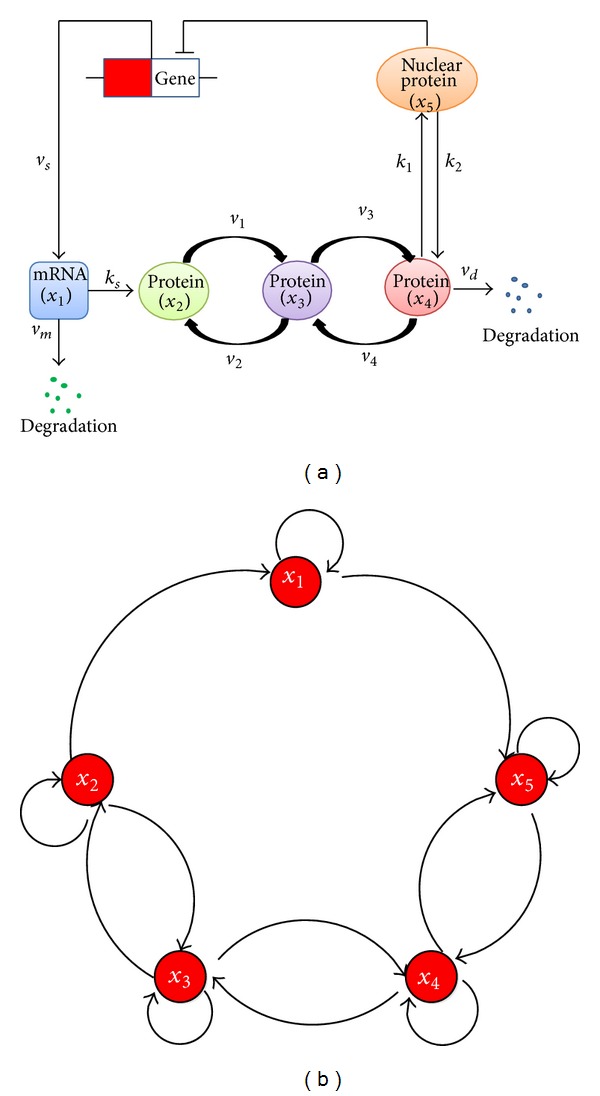
(a) A core model for circadian rhythms [7]. Parameters marked on the directed edges denote reaction rates. *x*_1_ denotes the concentration of the mRNA; *x*_2_, *x*_3_, *x*_4_, and  *x*_5_ are the concentrations of four forms of the protein expressed by the gene; *x*_2_, *x*_3_, and  *x*_4_ are cytosolic proteins; *x*_5_ is nuclear protein. The circadian gene transcripts mRNA *x*_1_ and then translates protein *x*_2_. Proteins *x*_2_, *x*_3_, and  *x*_4_ can be reversibly phosphorylated successively. *x*_4_ can be transported into the nucleus; the nucleus protein *x*_5_ can negatively regulate the expression of the circadian gene. The mRNA and protein *x*_4_ suffer from degradations. (b) The inference diagram [4] for the circadian rhythm model (3). If *x*_*i*_ appears in *x*_*j*_'s differential equation, then there will be a directed edge from *x*_*j*_ to *x*_*i*_.

The core circadian rhythm network is reported by Gonze et al. [7]; the detailed biochemical processes are shown in Figure 1(a). Figure 1(a) represents a prototype for the molecular mechanism of circadian oscillations based on negative autoregulation of gene expression. Real-world circuits corresponding to Figure 1(a) include the* per* mRNA and* PER* protein in* Drosophila* [11, 12] and* frq* mRNA and* FRQ* protein in* Neurospora* [13].

The core model involves gene transcription and transport of mRNA *x*_1_ into the cytosol where it is translated into protein *x*_2_ and degraded. Protein *x*_2_ can be reversibly phosphorylated from the form *x*_2_ into the forms *x*_3_ and *x*_4_, successively. The phosphorylated protein *x*_4_ is degraded or transported into the nucleus, and the nucleus protein *x*_5_ can negatively regulate the expression of its gene. Based on the work from Gonze et al. [7] in 2002, the modified mathematical model for the core circadian model can be established as follows. Consider

(3) dx1dt=vsKInKIn+x5n−vmx1Km+x1+u(t),dx2dt=ksx1−v1x2K1+x2+v2x3K2+x3,dx3dt=v1x2K1+x2−v2x3K2+x3−v3x3K3+x3+v4x4K4+x4,dx4dt=v3x3K3+x3−v4x4K4+x4−vdx4Kd+x4−k1x4+k2x5,dx5dt=  k1x4−k2x5,

where *x*_*i*_  (*i* = 1,…, 5) are state variables, which correspond to species concentrations of the mRNA, the four forms of proteins. *u*(*t*) represents the external input, which can be seen as the effect of the environment on the system. *K*_*i*_  (*i* = *I*, *m*, 1,2, 3,4, *d*), *k*_*j*_  (*j* = 1,2, *s*), and  *v*_*k*_  (*k* = *s*, *m*, 1,2, 3,4, *d*) are Michaelis constants, first-order reaction rate constants, and maximum rates of protein degradation, transcription, and phosphorylation. It is noted that if we set *u*(*t*) = 0, then system (3) degenerates into the model investigated in [7]. A typical set of parameter values for system (3) are shown in Table 1. Under the parameter values as shown in Table 1 and for *u*(*t*) = 0, dynamical system (3) can display circadian rhythms with a period close to 24 hours.

## 3. Observer Design for the Circadian Rhythm Model

For simplicity, in the following, we rewrite system (3) as the following form:

(4) $$\begin{array}{c} \frac{dx}{dt}=Ax\left(t\right)+f\left(x\left(t\right),u\left(t\right)\right),\\ y\left(t\right)=Cx\left(t\right), \end{array}$$

where *x*(*t*) = (*x*_1_(*t*),…, *x*_5_(*t*))^*T*^  and  *f*(*x*(*t*), *u*(*t*)) ∈ *R* ^5^ denotes the nonlinear term. *y*(*t*) denotes the monitored output. *C* is a constant matrix. *Ax*(*t*) denotes the linear term, with

(5) $$\begin{array}{c} A=\left[\begin{array}{ccccc} 0 & 0 & 0 & 0 & 0\\ k_{s} & 0 & 0 & 0 & 0\\ 0 & 0 & 0 & 0 & 0\\ 0 & -k_{1} & 0 & 0 & k_{2}\\ 0 & k_{1} & 0 & 0 & -k_{2} \end{array}\right]. \end{array}$$

For system (4), similar to the works in [4–6], our objective is to design the following observer, which can track the states of system (4):

(6) $$\begin{array}{c} \frac{dz}{dt}=Az\left(t\right)+f\left(z\left(t\right),u\left(t\right)\right)+K\left(y\left(t\right)-Cz\left(t\right)\right), \end{array}$$

where *K* is a gain matrix, which is to be determined. The estimation error dynamics are then given by

(7) $$\begin{array}{c} \frac{de}{dt}=\left(A-KC\right)e\left(t\right)+\left[f\left(x\left(t\right),u\left(t\right)\right)-f\left(z\left(t\right),u\left(t\right)\right)\right], \end{array}$$

where *e*(*t*) = *x*(*t*) − *z*(*t*).

From Liu et al. [4], the observability of a dynamical system can be revealed by its inference diagram. The inference diagram of system (3) is shown in Figure 1(b), where the five nodes are strongly connected and consist of the single root SCC. From the conclusion in [4], system (3) is observable, and one should only monitor any one of the five nodes in Figure 1(b). In the following, for simplicity, we assume that only *x*_1_(*t*) is monitored. The designed observer of system (3) is described as

(8) dz1dt=vsKInKIn+z5n−vmz1Km+z1+u(t)+kc(x1−z1),dz2dt=ksz1−v1z2K1+z2+v2z3K2+z3,dz3dt=v1z2K1+z2−v2z3K2+z3−v3z3K3+z3+v4z4K4+z4,dz4dt=v3z3K3+z3−v4z4K4+z4−vdz4Kd+z4−k1z4+k2z5,dx5dt=  k1z4−k2z5,

where *k*, *c* are the nonzero elements in matrix *K* and *C* of (6). For simplicity, we denote

(9) $$\begin{array}{c} g\left(K_{j},x_{i}\right)=\frac{x_{i}}{K_{j}+x_{i}}. \end{array}$$

Then,

(10) $$\begin{array}{c} \frac{K_{I}^{n}}{K_{I}^{n}+x_{5}^{n}}=1-g\left(K_{I}^{n},x_{5}^{n}\right). \end{array}$$

The corresponding error dynamics are described as

(11) de1dtvs[g(KIn,z5n)−g(KIn,x5n)] −vm[g(Km,x1)−g(Km,z1)]−kce1,de2dtkse1−v1[g(K1,x2)−g(K1,z2)] +v2[g(K2,x3)−g(K2,z3)],de3dtv1[g(K1,x2)−g(K1,z2)] −v2[g(K2,x3)−g(K2,z3)] −v3[g(K3,x3)−g(K3,z3)] +v4[g(K4,x4)−g(K4,z4)],de4dtv3[g(K3,x3)−g(K3,z3)] −v4[g(K4,x4)−g(K4,z4)] −vd[g(Kd,x4)−g(Kd,z4)]−k1e4+k2e5, de5dt =k1e4−k2e5.

Before we analyze the stability of system (11), we note that since *x*_*i*_, *z*_*i*_ represent species concentrations, they must be nonnegative and bounded [24]. Furthermore, since *g*(*K*_*j*_, *x*_*i*_) is continuous and differentiable, by the mean value theorem, there must exist *ξ*_*i*_ between *x*_*i*_ and *z*_*i*_, satisfying

(12) g(Kj,xi)−g(Kj,zi)=g′(Kj,ξ)(xi−zi)=Kj(Kj+ξi)2(xi−zi).

For system (11), we construct the following Lyapunov function:

(13) $$\begin{array}{c} V\left(t\right)=\frac{1}{2}\overset{5}{\underset{i=1}{\sum }}e_{i}{\left(t\right)}^{2}. \end{array}$$

Based on (12), the derivative of (13) along system (11) is

(14) V˙(t)=∑i=15ei(t)e˙i(t)=−[kc+VmKm(Km+ξ1)2]e12−V1K1(K1+ξ2)2e22 −[V2K2(K2+ξ3)2+V3K3(K3+ξ3′)2]e32 −[V4K4(K4+ξ4)2+VdKd(Kd+ξ4′)2+k1]e42 −k2e52+kse1e2+[V2K2(K2+ξ3)2+V1K1(K1+ξ2)2]e2e3 +(k1+k2)e4e5 +[V4K4(K4+ξ4)2+V3K3(K3+ξ3′)2]e3e4−nVsKInξ5n−1(KIn+ξ5n)2e1e5=eTQe.

Here,

(15) $$\begin{array}{c} Q=\left[\begin{array}{ccccc} \frac{-V_{m}K_{m}}{{\left(K_{m}+\xi_{1}\right)}^{2}}-kc & \frac{k_{s}}{2} & 0 & 0 & \frac{-nV_{s}K_{I}^{n}\xi_{5}^{n-1}}{2{\left(K_{I}^{n}+\xi_{5}^{n}\right)}^{2}}\\ {}* & \frac{-V_{1}K_{1}}{{\left(K_{1}+\xi_{2}\right)}^{2}} & \frac{0.5V_{2}K_{2}}{{\left(K_{2}+\xi_{3}\right)}^{2}}+\frac{0.5V_{1}K_{1}}{{\left(K_{1}+\xi_{2}\right)}^{2}} & 0 & 0\\ 0 & * & \frac{-V_{2}K_{2}}{{\left(K_{2}+\xi_{3}\right)}^{2}}-\frac{V_{3}K_{3}}{{\left(K_{3}+\xi_{3}^{\prime }\right)}^{2}} & \frac{0.5V_{4}K_{4}}{{\left(K_{4}+\xi_{4}\right)}^{2}}+\frac{0.5V_{3}K_{3}}{{\left(K_{3}+\xi_{3}^{\prime }\right)}^{2}} & 0\\ 0 & 0 & * & \frac{-V_{4}K_{4}}{{\left(K_{4}+\xi_{4}\right)}^{2}}-\frac{V_{d}K_{d}}{{\left(K_{d}+\xi_{4}^{\prime }\right)}^{2}}-k_{1} & \frac{k_{1}+k_{2}}{2}\\ {}* & 0 & 0 & * & -k_{2} \end{array}\right], \end{array}$$

which is a symmetrical matrix. *ξ*_*i*_, *ξ*_*i*_′ are values between *x*_*i*_ and *z*_*i*_. For appropriate gain *k*, if *Q* < 0, $\dot{V}(t)<0$. System (11) will be globally asymptotically stable. In other words, system (3) can be observed through the observer (8).

**Remark 1 .**

If any one of the other variables is used to track the state of the original system (3), one should only slightly revise the observer (8). If more than one variable is measured to track the original system, the observer can be similarly designed. For example, if *y*(*t*) in (4) is *y*(*t*) = (*c*_1_ *x*_1_,*c*_2_ *x*_2_)^*T*^, the control gain matrix *K* is chosen as

(16) $$\begin{array}{c} K=\left[\begin{array}{cc} \alpha & 0\\ 0 & \beta \\ 0 & 0\\ 0 & 0\\ 0 & 0 \end{array}\right]. \end{array}$$

Then, the observer is designed as

(17) dz1dt=vsKInKIn+z5n−vmz1Km+z1+u(t)+αc1(x1−z1),dz2dt=ksz1−v1z2K1+z2+v2z3K2+z3+βc2(x2−z2),dz3dt=v1z2K1+z2−v2z3K2+z3−v3z3K3+z3+v4z4K4+z4,dz4dt=v3z3K3+z3−v4z4K4+z4−vdz4Kd+z4−k1z4+k2z5,dx5dt=k1z4−k2z5.

For appropriate parameters *α*, *β*, one can easily prove that the original system is also observable from the observer (17). Obviously, the corresponding observer (17) is more complex than the observer (11).

**Remark 2 .**

There are many methods to prove the stability of a dynamical system. One can easily prove that the nonlinear terms on the right-hand side of system (11) are Lipschitz. For Lipschitz nonlinear systems, Rajamani [5] proposed a general theorem for the observer design. However, due to the complexity of the biological model, the theorem obtained in [5] fails to work for the error system (11). Therefore, we have used the mean value theorem to simplify the error system (11) and obtained a sufficient condition for the observer design. The matrix *Q* relies on the bound of system (3).

## 4. Numerical Simulations

**Figure 2:**
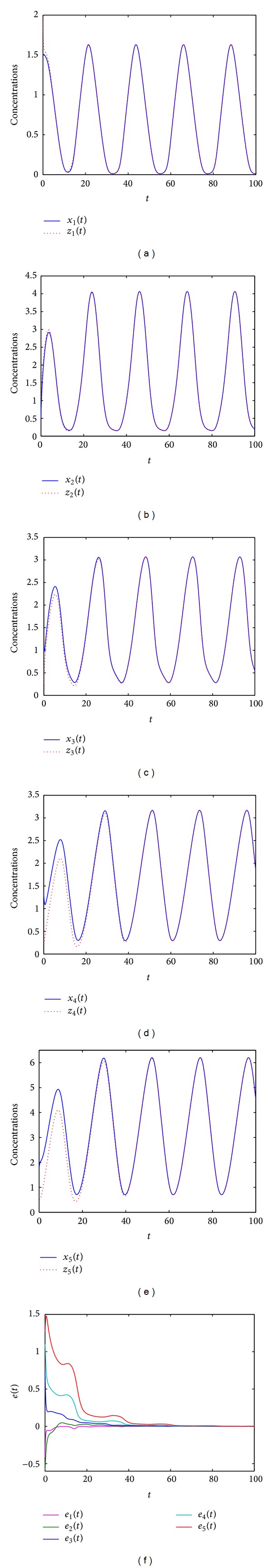
(a)–(e) State trajectories of systems (3) and (8). (f) The error dynamics of system (11). Here, *u*(*t*) = 0, *y*(*t*) = *x*_1_(*t*), and  *k* = 5. Initial values are randomly chosen.

Hereinafter, we numerically verify the effectiveness of the designed observers. Firstly, we assume *u*(*t*) = 0; the output function *y*(*t*) = *x*_1_(*t*). For *k* = 5, all the state variables in the original system (3) can be tracked by the observer (8). Under randomly initial values, Figure 2 shows the state trajectories of systems (3) and (8), as well as the error dynamics of system (11). From Figure 2, one can see that the state variables of (3) oscillate with a period close to 24 hours. The observer system (8) can track the states of the original system. The error between the observer system and the original system quickly approximates to zero.

**Figure 3:**
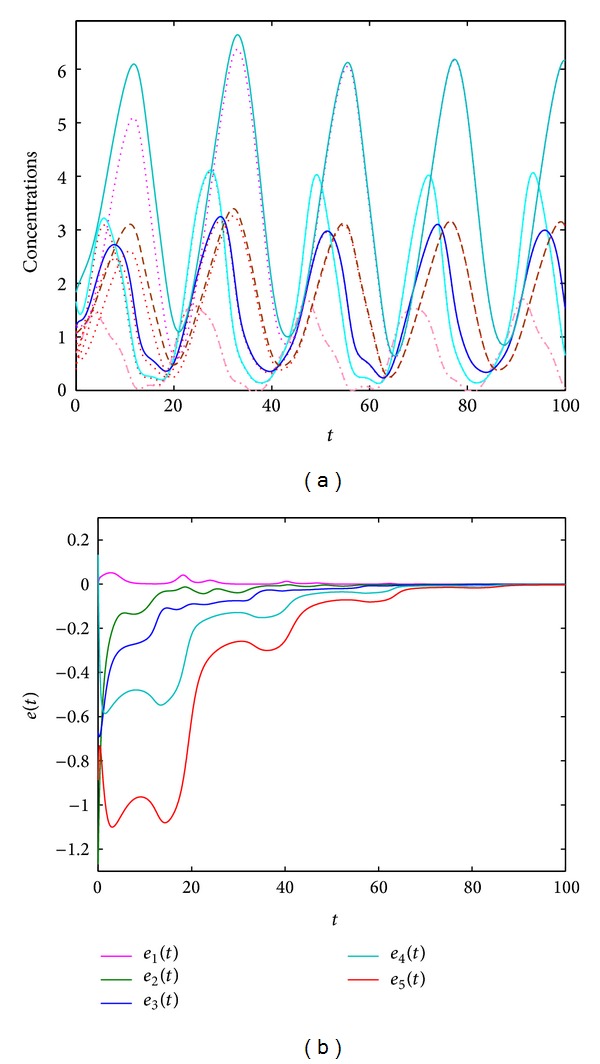
(a) State trajectories of systems (3) and (8) with *u*(*t*) = *ϵ*sin(*t*). (b) The error dynamics of system (11). Here, *y*(*t*) = *cx*_1_(*t*), *k* = 2.5, *c* = 2, and  *ϵ* = 0.1.

**Figure 4:**
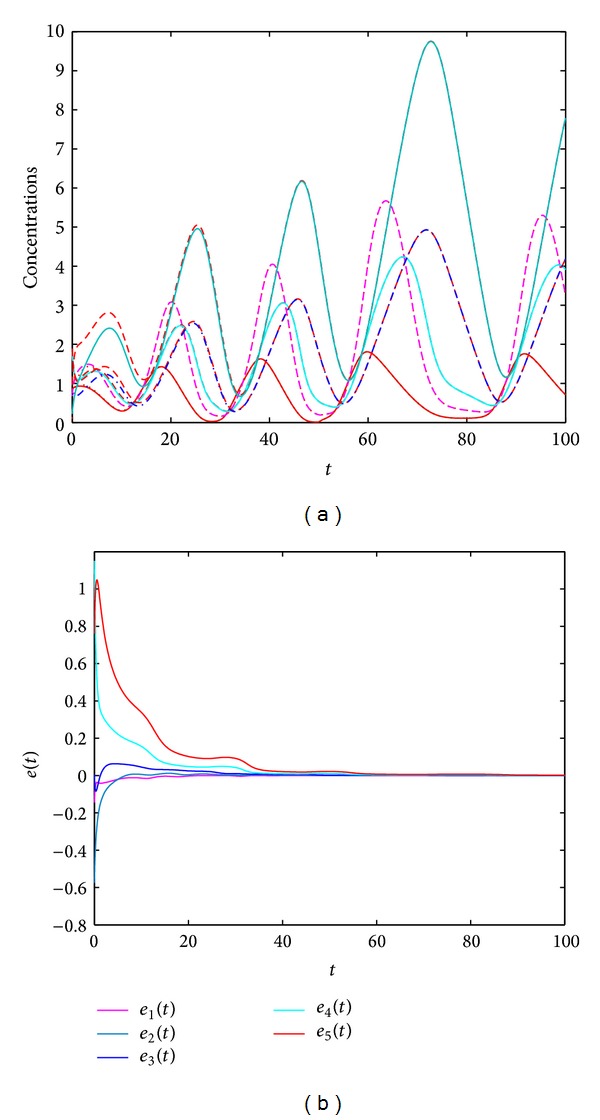
(a) State trajectories of systems (3) and (8) with step input signal *u*(*t*). (b) The error dynamics of system (11). Here, *y*(*t*) = *cx*_1_(*t*), *k* = 2.5, *c* = 2, and  *u*(*t*) = 0 for *t* ≤ 50 and *u*(*t*) = 0.1 for *t* > 50.

For different input signals and under appropriate gain *k*, system (8) can always track the states of system (3). For example, when *u*(*t*) = *ϵ*sin(*t*), *y*(*t*) = *cx*_1_(*t*), *k* = 2.5, *c* = 2, and  *ϵ* = 0.1, Figure 3 shows the state trajectories of systems (3) and (8) as well as the error dynamics of system (11). From Figure 3, we can see that the states of system (3) can be observed by the observer (8). The error system (11) converges to zero quickly. When *u*(*t*) is a step signal, the observer (11) can also well monitor the states of the original (3).  Figure 4 shows the case for *y*(*t*) = *cx*_1_(*t*), *k* = 2.5, and  *c* = 2, and the step input signal

(18) $$\begin{array}{c} u\left(t\right)=\{\begin{array}{cc} 0, & t\leq 50,\\ 0.1, & t>50. \end{array} \end{array}$$

For the cases discussed in Remark 1, when we choose *α* = 2, *β* = 3, *c*_1_ = 2.2, *c*_2_ = 1, and the following input signal,

(19) $$\begin{array}{c} u\left(t\right)=\{\begin{array}{cc} 0, & t\leq 50,\\ 0.2, & 50<t<150,\\ 0, & t\geq 150, \end{array} \end{array}$$

**Figure 5:**
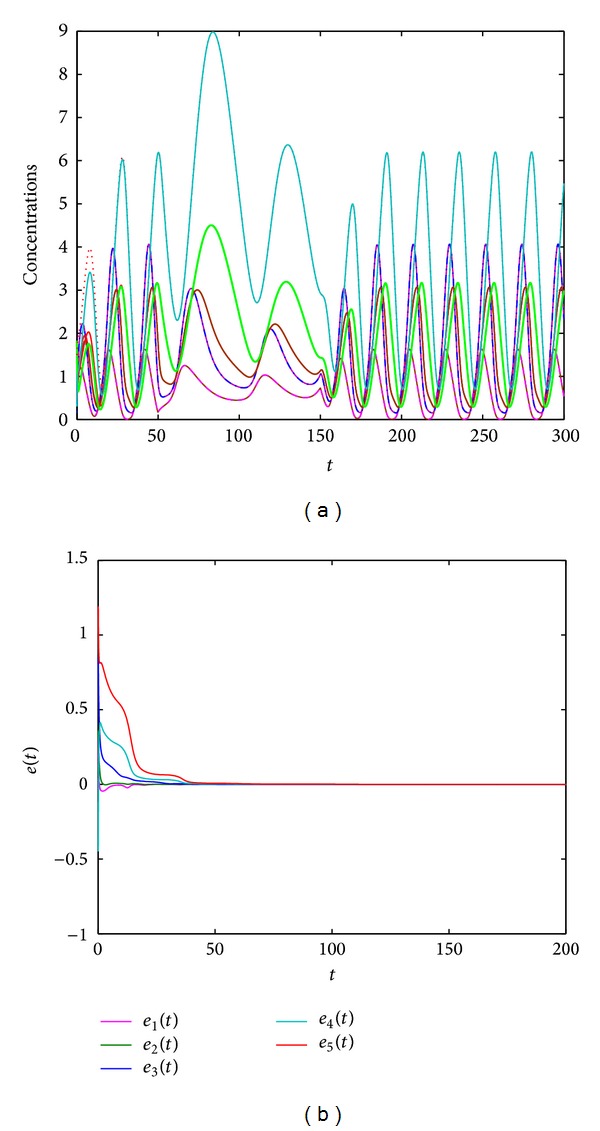
(a) State trajectories of systems (3) and (17) with step input signal *u*(*t*) in (19). (b) The error dynamics between systems (3) and (17). Here *y*(*t*) = (*c*_1_ *x*_1_(*t*), *c*_2_ *x*_2_(*t*))^*T*^, *α* = 2, *β* = 3, *c*_1_ = 2.2, and  *c*_2_ = 1.

Figure 5 shows the numerical simulation results for such case. From Figure 5, we see that the observer (17) can also well track the states of system (3). Additionally, combined with the simulation results as shown in Figures 2–5, we can conclude that the designed observers have good performance under various kinds of inputs, and the input signal *u*(*t*) can affect the period of the circadian oscillator.

## 5. Discussions and Conclusions

Biological systems are typical complex dynamical systems. To efficiently infer the state of a biological system, it is necessary to develop some simple observers via monitoring a few system variables. Based on the recently proposed graphical approach, we have designed some rather simple observers for a core circadian rhythm network. For various input signals and under appropriate control gains, the designed observer can well infer the states of the original system. The investigations in this paper further support the conclusions in [4]. Real-world applications of the related investigations on biological networks include the experimental design and control of the related biological systems.

We have considered three types of inputs, and it is intriguing to investigate the observer design problems for stochastic systems, since biological systems are inherent stochastic and perturbed by environment [14–16]. Another question that deserves to be further investigated is to develop some general theorems to guarantee the observability of the biological systems [25–28]. Finally, it is also intriguing to investigate the observability of large-scale biological networks [17], such as the yeast cell cycle network with boolean dynamical model or differential equation models [18–23]. These topics will be discussed in our future works.

## Figures and Tables

**Table 1:** Parameter values.

Parameter	Value
*v*_*s*_	0.5 nMh^−1^
*n*	4
*K*_*m*_	0.2 nM
*v*_1_	6.0 nMh^−1^
*v*_2_	3.0 nMh^−1^
*v*_3_	6.0 nMh^−1^
*v*_4_	3.0 nMh^−1^
*v*_*d*_	1.5 nMh^−1^
*k*_1_	2.0 h^−1^
*K*_*I*_	2 nM
*v*_*m*_	0.3 nMh^−1^
*k*_*s*_	2.0 h^−1^
*K*_1_	1.5 nM
*K*_2_	2.0 nM
*K*_3_	1.5 nM
*K*_4_	2.0 nM
*K*_*d*_	0.1 nM
*k*_2_	1.0 h^−1^
